# Integrating HIV care and treatment into primary healthcare: Are clinics equipped?

**DOI:** 10.4102/phcfm.v6i1.616

**Published:** 2014-08-28

**Authors:** Talitha Crowley, Ethelwynn L. Stellenberg

**Affiliations:** 1 Division of Nursing, Faculty of Medicine and Health Sciences, Stellenbosch University, South Africa

## Abstract

**Background:**

The demand for HIV care and treatment services is increasing rapidly and strategies to sustain long-term care should be employed. The decentralisation and integration of HIV care and treatment services into primary healthcare (PHC) is vitally important in order to ensure optimal access to life-saving antiretroviral therapy and ongoing chronic care. Conversely, the PHC system is fraught with the current burden of disease.

**Setting:**

The study was conducted in PHC clinics in the uMgungundlovu district, Kwa-Zulu Natal.

**Aim:**

The objectives of the study were to assess whether PHC clinics were equipped to deliver integrated HIV services and to evaluate the availability of resources as well as support systems for HIV care and treatment in PHC clinics.

**Methods:**

A quantitative, cross-sectional descriptive study was undertaken in 20 randomly-selected, eligible clinics in the uMgungundlovu district, KwaZulu-Natal, South Africa. An evaluation instrument was completed through observations and review of the clinic data records. Criteria were based on the World Health Organization's guide to indicators for antiretroviral programmes as well as South African HIV standards for PHC facilities.

**Results:**

None of the clinics were equipped adequately. Clinics with a higher patient load had poorer scores, whilst clinics providing antiretroviral therapy were better equipped in terms of human resources and infrastructure.

**Conclusion:**

HIV services are an essential part of primary healthcare and clinics need to be equipped adequately in order to render this service. It is unlikely that the over-burdened health system would be able to cope with an increased number of patients on antiretroviral therapy in the long term, whilst maintaining quality of services, without support being given to PHC clinics.

## Abstrait

**L'intégration des soins et du traitement du VIH dans les services de santé primaire: les cliniques sont-elle équipées pour cela?**

**Contexte:**

La demande des services de soins et traitement du VIH augmente rapidement; aussi faudrait-il employer des stratégies pour maintenir les soins à long terme (devraient être employées). La décentralisation et l'intégration de ces services dans les soins de santé primaires (SSP) sont (est) importantes pour assurer un accès optimal au traitement de sauvetage et aux soins continus.

**Objectifs:**

Evaluer si les cliniques de SSP étaient équipées pour offrir des services intégrés du VIH. Evaluer la disponibilité des ressources ainsi que des systèmes de soutien pour les soins et traitement du VIH.

**Méthodes:**

Une étude descriptive quantitative, transversale a été menée dans 20 cliniques (aléatoirement) sélectionnées au hasard dans le district d'uMgungundlovu, au KwaZulu-Natal, en Afrique du Sud. Un instrument d’évaluation a été complété par observation et revue des données. Les critères étaient fondés sur les indicateurs de programmes antirétroviraux de l'Organisation Mondiale de la Santé, et sur les normes sud-africaines pour (sur) le VIH.

**Résultats:**

Aucune des cliniques n’était correctement équipée. Les cliniques à (a) charge élevée de patients avaient les (plus pauvres) scores les plus bas; celles de thérapies antirétrovirales étaient mieux équipées en ressources humaines et infrastructures.

**Conclusion:**

Les services du VIH sont une partie essentielle des soins de santé primaires; les cliniques doivent être équipées pour rendre ce service. Il est peu probable que le système de santé surchargé sera (serait) en mesure de faire face à une augmentation du nombre de patients sous traitement antirétroviral à long terme, tout en maintenant la qualité des services, sans soutien apporté aux centres de SSP.

## Introduction

### Social value

There has been a significant expansion in the need for HIV care and treatment in Africa as well as in the world.^1^ Such a scale-up of treatment brings unique challenges that warrant investigation. One of the critical challenges is the delivery of long-term care for the increasing population of clients on antiretroviral therapy within the context of overburdened health systems.^2^

The global incidence of HIV seems to have stabilised, but the number of people receiving antiretroviral therapy has increased, with 6.65 million people receiving antiretroviral therapy by the end of 2010.^1^ With the introduction of the World Health Organization (WHO) Global Health Sector Strategy on HIV/AIDS, 2011–2015^3^ and the UNAIDS 2011–2015 Strategy: Getting to Zero,^4^ there is a renewed focus on sustainable models for the delivery of long-term HIV care and treatment. In order to promote accessible, equitable and sustainable HIV services within resource-constrained systems, HIV programmes need to be decentralised (brought closer to the people) and integrated with other health services.^2^ A primary healthcare (PHC) approach is generally considered as being central to providing equitable healthcare.^5–7^

### Scientific value

Current trends suggest increased support for the decentralisation of HIV treatment and care services, task shifting and the integration of these services within the PHC system.^2, 8^

The decentralisation of care includes the provision of HIV care and treatment such as antiretroviral therapy, both in PHC clinics and within communities.^9^ In general, task shifting is a characteristic of decentralisation. Tasks traditionally performed by higher cadres are shifted to lower cadres with less training such as non-physician clinicians, nurses, nurse-assistants, community healthcare workers and people living with HIV.^10^

‘Integration’ is described differently by various authors but, in broad terms, means services previously provided vertically which are now delivered as a package of care within mainstream health services.^6, 8^ The integration of HIV services into primary healthcare has the benefit of promoting coherent and holistic services. It increases access to care, allows more people to be treated, improves retention in care, saves costs and potentially leads to improved patient outcomes.^11–13^ However, the evidence for the benefits of decentralisation and integration of HIV care and treatment is not definitive and a number of studies found no difference in health-related outcomes or access to antiretroviral therapy.^6, 14, 15^

It has been found that the integration of HIV care and treatment services in primary healthcare benefit the larger PHC system.^12^ Nevertheless, ongoing challenges such as logistical and infrastructural constraints as well as adding tasks to overburdened staff are barriers to successful integration.^12, 16, 17^

It is apparent that the decentralisation and integration of HIV care and treatment into primary healthcare has potential benefit, yet there are few clear recommendations for effective integration of HIV care and treatment into primary healthcare.^15^ This study describes the evaluation of PHC clinics within a health district in South Africa with a high HIV burden, to establish whether these clinics are equipped for integrated HIV care and treatment services.

Given the potential benefit of the integration of HIV care and treatment services to improve access to care and provide continuity of long-term care, it is crucial to identify available resources in PHC clinics that will enable quality HIV care and treatment services. Furthermore, quantifiable methods to assess whether clinics are equipped fully for integration of care could be beneficial with regard to measuring progress.

### Conceptual framework

**FIGURE 1:**
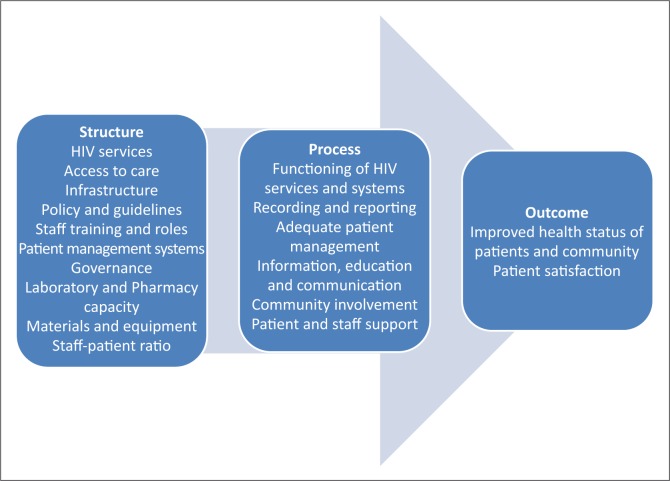
Conceptual framework indicating indicator clusters.

The conceptual framework for the study was based on Donabedian's model for healthcare quality evaluation,^18^ which constitutes of structure, process and outcome indicators. For the purpose of this study, the structure and process indicators according to the Donabedian model were applied in order to evaluate the objectives as determined for the study. Several indicators, grouped in clusters such as recording and reporting, physical space, basic HIV services rendered and laboratory capacity, were used to evaluate whether clinics were equipped for integrated services (Figure 1).

### Aim and objectives

The objectives of the study were to assess whether PHC clinics were equipped adequately for the delivery of integrated HIV services and to evaluate the availability of resources and support systems for HIV care and treatment in PHC clinics.

## Research method and design

### Study design

A quantitative cross-sectional descriptive design was used.

### Setting

The study was conducted in PHC clinics in the uMgungundlovu district, Kwa-Zulu Natal. Kwa-Zulu Natal has the highest HIV prevalence in South Africa (39.5%).^19^

### Study population and sampling strategy

The target population for the purpose of this study was provincial clinics and local government clinics of the uMgungundlovu District, KwaZulu-Natal. The eligibility criteria for choosing clinics were the following:

- Clinics were operational from a permanent location.
- Service delivery took place every week day for eight hours per day.
- A comprehensive PHC service was provided.

The target population for the purpose of this study was 47 clinics. A simple random sample of 20 (43%) of the clinics was drawn in order to ensure representation of the target population. The sample was selected by assigning numbers to the clinics on the list obtained from the district office. Numbers were selected randomly by a third party to ensure that each clinic had an equal chance of being selected.

### Data collection

The researcher collected all data with the use of an instrument. A new instrument based on the literature, the WHO's guide to indicators for monitoring and evaluating national antiretroviral programmes^20^ and HIV standards for PHC facilities in South Africa,^21^ was developed by the researcher. The instrument was divided into Section A, which included demographical data about the clinic, and Section B, which contained indicator clusters such as: recording and reporting; physical space; basic HIV services rendered; laboratory capacity; and pharmacy capacity (Figure 1).

#### Pilot test

Pilot testing of the instrument was conducted in two clinics that were not part of the sample. It was identified during the pilot test that the instrument only allowed for dichotomous responses. During data collection, it was observed that many of the indicators should rather be measured categorically and the instrument was consequently adapted. Clinics were given a score out of ‘3’ for each of the 136 indicators, where a score of ‘3’ was allocated if all requirements were met; ‘2’ for most requirements met; ‘1’ for few requirements met; and ‘0’ for no requirements met. A minimum acceptable score of 80% was decided upon, including meeting the critical criteria, for clinics to be equipped adequately to deliver integrated HIV care and treatment services. Critical criteria were indicators that were regarded as essential for effective functioning and their selection was based on the literature. These 87 critical points included indicators such as whether the clinic had 12 months of patient data available, the implementation of infection prevention and control procedures and the provision of basic HIV services, such as HIV testing and counselling. Data from the pilot test were not included in the main study.

#### Validity and reliability

The face and content validity of the instrument were strengthened by pilot testing the instrument and consultations with experts in HIV systems research, nursing research and a qualified statistician. Detailed field notes were taken of observations made and information from clinic data records so as to promote accurate scoring.

#### Main study

Data for the main study were collected from clinic data records and from observations made by the researcher from December 2008 to January 2009. Clinic managers were consulted when relevant data could not be found.

### Data analysis

Data were entered into Microsoft Excel by the researcher and a statistical analysis program, STATISTICA (Release 8), was used to analyse the data. Comparisons between variables such as clinic demographical data, indicators and the overall clinic score were made by using appropriate techniques, such as Fisher exact, Mann-Whitney U and Spearman correlations. These techniques are all used on smaller sample sizes in order to ensure the accurate interpretation of results. A 5% significance level (*p* < 0.05) was used as a guideline to determine significant relationships.

### Ethical considerations

Ethical approval for the study was obtained from the Health Research Ethics Committee at the Faculty of Medicine and Health Sciences, University of Stellenbosch (N08/09/237). Permission to conduct the research was obtained from the KwaZulu-Natal provincial research committee, the uMgungundlovu district manager and local municipality managers. Written informed consent was obtained from clinic managers before data collection. The interruption of clinic procedures was limited since the researcher obtained data from clinic data registers and observations. Clinic names were coded and data de-identified in order to protect anonymity.

## Results

### Clinic demographical data

The ratio between provincial and local government clinics was 3:2 (Table 1). Most clinics (*n* = 13; 65%) were open on all week days for eight hours per day, six clinics (30%) were open for 24 hours for seven days per week and one clinic (5%) was open on weekdays and on Saturdays. Non-governmental organisations (NGOs) employed staff for the antiretroviral therapy (ART) programme in eight (40%) clinics. Statistical analysis revealed that clinics that had NGO support for the HIV programme were more likely to provide ART as part of their HIV care and treatment services (Fisher exact *p* < 0.01).

**TABLE 1:** Demographical data of clinics (*n* = 20).

Variable	*n*	%
Local government (municipality)	8	40
Provincial government	12	60
Clinics with NGO support for HIV programme	8	40
Clinics with no NGO support for HIV programme	12	60
Clinics where ART was available	10	50
Clinics where ART was not available	10	50

*n*, number of clinics; NGO, non-governmental organisation; ART, antiretroviral therapy.

### Indicator clusters

Recording and reporting were assessed by evaluating whether clinics had a complete set of data available for the 12 months prior to the study. Only 11 clinics (55%) had complete data available for the primary healthcare head count, HIV counseling and testing (HCT) and TB monitoring.

Physical space comprised storage space for medical supplies as well as consultation rooms. Only six (30%) clinics had sufficient storage space for nutritional supplements and medication. In 10 (50%) of the clinics, the number of consultation rooms was not sufficient for the current purposes. Sharing of consultation rooms between healthcare workers was observed to be a common practice, posing concerns for patient privacy. Clinics that provided ART had more consultation and counseling rooms available than clinics where ART was not provided (Mann-Whitney U *p* < 0.01).

Adequate referral systems are central to the functioning of decentralised ART services. Reliable transport systems for patient referral were only available in eight (40%) clinics, whilst two (10%) clinics did not have a dedicated telephone or cellphone line in order to communicate with referral facilities. With regard to patient information, education and communication (IEC), HIV educational material in local languages was only available in seven (35%) of the clinics.

Basic HIV services included indicators such as the provision of family planning, sexually-transmitted infection management and HIV counseling and testing (HCT). All of the basic HIV services were available and utilised, with half (50%) of the clinics proving ART and 15 (75%) clinics preparing patients for ART initiation, including antiretroviral adherence preparation and baseline blood investigations. All of the clinics (100%) provided HCT and CD4 blood testing, although provider-initiated counseling and testing (PICT) was only practised in five (25%) clinics. At the time of the study, professional nurses did not initiate patients on ART and in 19 (95%) of the clinics, less than half of the staff members had received any HIV-related training. In two (10%) of the clinics, it was observed by the researcher that there were no dedicated professional nurses for managing patients on ART and patients collected medication from untrained staff, such as enrolled nurses. There was no significant difference between clinics that did and did not provide ART with regard to staff trained in HIV management (Mann-Whitney U *p* = 0.47).

Adherence to the policies and national treatment guidelines or protocols is important for ensuring safe patient care. Updated policies and guidelines that were organised in a file could only be located in two (10%) of the clinics. The implementation of universal infection precautions were found to be challenging in three clinics (15%) since there were no handwashing basins or alcohol-based hand disinfectant and a lack of sharp containers and medical waste bins.

Patient support was found to be lacking as nine clinics (45%) did not have functional support groups for HIV-positive patients on-site and three (30%) clinics providing ART did not have a system in place for tracing clients that default on their treatment. However, clinics providing ART were more likely to have a functional support group for people living with HIV (Mann-Whitney U *p* = 0.01).

Community involvement, defined as being any evidence of interaction between the clinic and community-based organisations or the provision of community services, was absent in 18 (90%) of the clinics. Of the clinics that engaged with the community, one clinic (5%) interacted with traditional healers in the community to support patients on ART to adhere to therapy and one clinic (5%) had a record of community health workers (CHWs) who were active in the community for referral of patients for home-based or palliative care. None of the clinics had a social worker on-site and none of the clinics had a record of referring patients for home-based or palliative care.

Laboratory and pharmaceutical services are both essential components of the provision and monitoring of ART. Adequate laboratory services were available in more than half (*n* = 15; 75%) of the clinics. However, only eight (40%) of the clinics had a turn-around time of less than two weeks on certain blood results and two (10%) of the clinics experienced problems with the system for transport of specimens to the laboratory. The laboratory system could also not be accessed electronically in any of the clinics.

Dispensaries (dedicated medicine rooms) were used for storing medication in 19 (95%) of the clinics and one clinic (5%) used cupboards in the patient consultation rooms. Insufficient storage space for storing medication existed in half (50%) of the dispensaries. These clinics did not have sufficient shelf space for medication. There were no dedicated storage areas and boxes containing medication or medical supplies were stacked on the floors of consultation rooms and dispensaries. Adequate security for the dispensary was only available in 12 (60%) of the clinics. In four (20%) of the clinics, the room where the medication was kept was not air-conditioned as the air-conditioner was broken at the time. All the clinics had essential medicines available, although functional stock-outs of essential drugs were reported by clinic managers in 18 (80%) of the clinics.

With regard to governance and management, only three clinics (15%) were supervised every three months by the district HIV/AIDS coordinator. Provincial government clinics were significantly more likely to be visited every three months by the HIV/AIDS coordinator than local government clinics (Mann-Whitney U *p* < 0.01). The latter finding could suggest communication challenges between the HIV/AIDS coordinator and local government clinics.

In nine (45%) of the clinics, the clinic building was not adequately maintained. Problems observed by the researcher with regard to maintenance were numerous and included: walls requiring painting, leaking roofs, ‘rotten’ ceilings that were falling down, sewerage systems that were out of order, poor air-conditioning and leaking toilets. There was lack of electricity or water supply in some of the park homes outside the clinics that were utilised for HIV services. Basic equipment such as otoscopes, thermometers, blood pressure monitors and measuring jugs for mixing paediatric medication was missing or not functioning properly in two (10%) of the clinics.

Average monthly patient statistics were calculated where clinics had at least three months of data available (Table 2). There were large variations in the PHC monthly headcount, which could be attributed to the fact that some clinics rendered a 24-hour service and some only an eight-hour daily service. Some of the rural clinics did not have a very high patient headcount. Furthermore, there could be differences in how the clinics counted and documented their PHC headcount.

**TABLE 2:** Clinic statistics.

Average monthly statistic	*n*	Mean	Standard deviation	Minimum	Maximum
PHC headcount	20	3850.55	2397.33	959	9438
HCTs done	20	124.05	69.99	27	301
CD4 counts done	18	74.06	57.82	15	239
ART up-referrals	16	17.31	12.76	2	40
ART follow-ups	9	225.44	199.09	21	558
Patients on waiting list for ART	10	42.6	66.6	4	229

*n*, number of clinics; PHC, primary healthcare; HCT, HIV counseling and testing; CD4, cluster of differentiation 4; ART, antiretroviral therapy.

Clinics where ART was provided reflected a significantly higher PHC headcount than clinics that did not provide ART (Mann-Whitney U *p* < 0.01).

The average number of patients seen per staff member (Registered Nurse, Enrolled Nurse and Enrolled Nurse Assistant) per day was 22.77 (SD = 9.87), with a minimum of 9.76 and a maximum of 49.94.

Clinics with higher PHC headcounts had significantly more staff members than clinics with lower headcounts (Spearman *r* = 0.87, *p* < 0.01). Furthermore, clinics that provided ART had significantly more staff compared with clinics that did not provide ART (Mann-Whitney U *p* < 0.01). The provision of ART as part of HIV care and treatment services was therefore associated with increased PHC headcounts and the employment of more staff members.

### Clinic score

Although five (25%) clinics obtained an average clinic score percentage of more than 80%, none of the clinics met all critical points. The high mean of 73.98 (SD = 6.93) for the total score signifies that PHC clinics have the potential to provide quality integrated HIV treatment and care services should they receive adequate support.

The clinic scores were affected significantly by the number of staff members as well as the patient load (number of patients seen by each nursing staff member per day) of the clinic. Clinics with more staff members were more likely to achieve a high score for the instrument than clinics with less staff members (Spearman *r* = 0.71, *p* < 0.01). Furthermore, the higher the average number of patients seen by each staff member per day (patient load), the lower the average score (Spearman *r* = -0.63, *p* < 0.01). This may denote the importance of sufficient human resources to ensure quality of services.

Clinics where an NGO was providing additional resources for the ART programme obtained a significantly higher score than clinics where there was no NGO involvement (Mann-Whitney U *p* = 0.01). NGO support for the ART programme in the form of human and other resources may therefore improve PHC capacity for HIV care and treatment services.

## Discussion

### Key findings

The results indicated that most basic HIV care and treatment services were provided in PHC clinics. However, PHC clinics were not equipped adequately to render integrated HIV care and treatment services. This was because of a lack of capacity on both a clinical and health system level. Although half of the clinics already provided ART, the provision of ART is not the primary indicator for determining whether clinics are equipped to render quality integrated services. Key elements such as recording and reporting, human resource constraints, physical space and support systems needed to be addressed.

Aspects of HIV care and treatment such as the availability of physical space, trained healthcare professionals, updated guidelines, essential drugs, nutritional support, support groups and tracing of defaulters were found to be lacking. These findings are consistent with studies conducted in other provinces in South Africa.^16, 17^

Clinics that provided ART had more staff available. The average monthly ART patient headcount for these clinics was 225.44 (SD = 199.09). The ART rollout would inevitably increase the patient load of clinics and this was confirmed in the results. If human and other resources do not increase, other initiatives for managing patients on ART in the long term need to be explored. Workload and capacity constraints as well as logistical and infrastructural challenges and a lack of supervision and support were ongoing challenges that were documented in other studies.^8, 16, 17, 22^

### Discussion of key findings

Integrating additional services into primary healthcare without providing the adequate human resources and ongoing support could compromise the quality of care provided to patients and have long-term repercussions for reaching strategic developmental and global health goals.

Essential HIV care and treatment services as well as managerial and infrastructural support are key components for ensuring quality services. The provision of ART at a PHC level should be supported with additional human resources, infrastructure and the implementation of comprehensive models for integration of care.

### Strengths and limitations

Limitations included that the study was conducted in one district within the context of a high HIV burden of disease and the results may therefore not be generalised to other settings. The number of clinics that was used was limited because of the depth of assessment in each of the clinics. The instrument and the scoring system used needs further testing in order to assess the reliability. Scores could have been calculated for individual structure and process indicator clusters which may prove beneficial in identifying specific areas that need improvement.

### Implications or recommendations

With the ongoing effort to promote nurse-led services, emphasis should be placed on evaluating individual clinics with regard to resources and management systems for integrating HIV care and treatment services. Certain organisational systems, protocols and resources should be in place in order to ensure quality of care and continuity of services. Districts and clinics may vary with regard to human resources and infrastructure, necessitating individualised interventions.

However, HIV resources have been found to rehabilitate the PHC infrastructure12 and a follow-up study is therefore necessary in order to evaluate the impact of the escalated ART rollout since 2010 on PHC clinics in South Africa.

## Conclusion

The shift from doctor-led hospital-based care to nurse-led primary healthcare necessitates careful consideration for patient safety and quality of care. The clinics evaluated in this study were not equipped adequately for integration of HIV care and treatment into PHC services. Integration of HIV care and treatment services into primary healthcare could have detrimental effects on the quality of long-term HIV care and treatment within the current overburdened PHC system.

With a renewed focus on the provision of primary healthcare and integration of HIV care and treatment in mainstream health services, additional resources and support should be allocated to PHC clinics in order to sustain long-term HIV care that is of a high quality and will not compromise other services. Such an approach will inevitably strengthen the larger PHC system in order to address the quadruple burden of disease in South Africa.
